# Winter arctic sea ice volume decline: uncertainties reduced using passive microwave-based sea ice thickness

**DOI:** 10.1038/s41598-024-70136-9

**Published:** 2024-09-09

**Authors:** Clement Soriot, Martin Vancoppenolle, Catherine Prigent, Carlos Jimenez, Frédéric Frappart

**Affiliations:** 1 LERMA, Observatoire de Paris, CNRS, Université PSL, Paris, France; 2 Centre for Eath Observation Science, University of Manitoba, Winnipeg, MB Canada; 3 Sorbonne Université, Laboratoire d’Océanographie et du Climat, CNRS/IRD/MNHN, Paris, France; 4 Estellus, Paris, France; 5 ISPA, UM 1391, INREA/Bordeaux SCience Agro, Villenave, d’Ornon 33140 France

**Keywords:** Climate sciences, Ocean sciences

## Abstract

Arctic sea ice volume (SIV) is a key climate indicator and memory source in sea ice predictions and projections, yet suffering from large observational and model uncertainty. Here, we test whether passive microwave (PMW) data constrain the long-term evolution of Arctic SIV, as recently hypothesized. We find many commonalities in Arctic SIV changes from a PMW sea ice thickness (SIT) 1992-2020 time series reconstructed with a neural network algorithm trained on lidar altimetry, and the reference PIOMAS reanalysis: relatively low differences in SIV mean (4615 km^3^, 37%), SIV trends (46 km^3^/yr, 17%), and phased variability (r^2^=0.55). Key to reduced differences is the consistent evolution of many SIV contributors: seasonal and perennial ice coverage, their SIT contrast, whereas perennial SIT provides the largest remaining uncertainty source. We argue that PMW includes useful SIT information, reducing SIV uncertainty. We foresee progress from sea ice reanalyses combining dynamical models and data assimilation of PMW SIT estimates, in addition to the already assimilated PWM sea ice concentration.

## Introduction

Arctic sea ice loss is attributed to anthropogenic CO_2_ emission^1,2^, a key element in favor of an anthropogenic origin of climate change^3^. Empirical knowledge of Arctic sea ice loss mostly comes from satellite Passive MicroWave (PMW) retrievals of sea ice fractional coverage^4,5^. Available since 1979, hence providing the longest continuous climate time series^3,6^, PMW records show a decrease in sea ice coverage in any month of the year since 1979^7,8^ exceeding 10% per decade during summer months^9^.

Sea Ice Thickness (SIT) provides a more in-depth characterization of sea ice changes than coverage. Indeed, SIT directly relates to the negative storage of thermal energy in sea ice. In addition, spatially integrated SIT determines the Sea Ice Volume (SIV). To date, compilation of observational sources and ice-ocean model reanalyses have established a significant SIT decrease above 60% of the mean in the Central Arctic between the 1970s and the 2010s^10,11^, along with a corresponding SIV loss^12–14^. Both findings are rated with *very high confidence* in recent IPCC assessment reports^7,8^. In Fram Strait, the primary gateway for Arctic sea ice, the longest consistent SIT record available confirms thinning, and reveals a regime shift in 2005-2007, marked by a rapid reduction in modal SIT, variance, and thick ice prevalence^15^.

Quantitatively however, uncertainties in Arctic SIV are considered to exceed 50% for the absolute value and 30% for the long-term trend^8,12,13,16,17^, for several reasons. First, *in situ* sources (e.g. airborne, submarine, drilling) inconsistently and unevenly sample SIT^10,18–21^. Furthermore, space-borne lidar and radar altimeter SIT retrievals cover less than two decades with noncontinuous observations^22,23^ and suffer from error propagation, mostly from uncertain snow depth^16,24–26^. Finally, ice-ocean model reanalyses, while benefiting from the assimilation of PMW Sea Ice Concentration (SIC) and Sea Surface Temperature (SST), are hindered by SIT model errors, resulting in a large SIV inter-model spread^12–14^.

PMW could provide an alternative, consistent observational source for SIT time series. Recent work has uncovered relationships between SIT and PMW signals not only for sea ice up to 50 cm - 1 m^27–30^, but also for thicker ice^31^. Building on this, several groups developed machine-learning based algorithms using PMW data, trained on lidar and/or radar satellite altimetry SIT data. These algorithms demonstrated comparable performance with standard altimetry when tested against independent airborne SIT observations^31–33^. The PMW SIT algorithm^31^ processes the few PMW channels available over several decades and therefore offers means to provide several decade-long of consistent SIT time series. On this basis, we produced and evaluated a new 1992-2020 SIT estimate and SIV volume PMW time series over the pan-Arctic for the cold month (October-March), with the^31^ algorithm. It is applied on a long time series of climate-quality satellite observations from Special Sensor Microwave / Imager (SSM/I) and Special Sensor Microwave Imager Sounder (SSMIS) (Fundamental Climate Data Record^34^).

In this work, we examine whether a new PMW SIT estimate records^31^ improves our knowledge of Arctic SIV and its long-term changes. To accomplish this, the new PMW SIT retrieval product is evaluated against the PIOMAS (Pan-Arctic Ice-Ocean Modelling and Assimilation System^35^) reanalysis. The PIOMAS SIT product has been thoroughly evaluated against *in situ* SIT observations^12^ and is considered as a reference. Similar to PIOMAS (and other modelling systems assimilating sea ice concentration), the new SIV time series benefits from the well-resolved ice-water contrast by PMW emissions, for the estimation of the sea ice concentration. A key distinction between PMW and PIOMAS lies in the fact that PMW SIT relies on PMW observations only, using a neural-network-based algorithm, while PIOMAS SIT derives from model calculation and data assimilation (see Methods), leading to different uncertainties. PIOMAS suffers from model biases stemming from improper model physics, numerics or calibration. The algorithm^31^ trained on 2018-2019 lidar altimetry data inherits altimetry issues, in addition to PMW sensitivity to snow melt in the spring, and has larger uncertainties in the earliest sections of the time series, particularly in the 1990s, when the thickest ice on record was present in the Arctic Basin. Nevertheless, we find the PMW and PIOMAS SIT and SIV time series over the last three decades similar in many respects, including changes in volume, thickness distribution, and the seasonality of these changes.

## Results

### PMW and PIOMAS similarly depict Arctic sea ice thinning and volume loss

SIV is the spatially-integrated SIT over sea ice area. To estimates SIV, two SIT datasets were utilized: the v2.1 PIOMAS reanalysis^35^ and a PMW SIT estimates^31^. The PIOMAS record is based on a simulation with a sea ice-ocean model assimilating passive microwave SIC and infrared SST records^12,35^. The PMW SIT data set is based on a neural network retrieval algorithm trained with SIT lidar altimetry data (ICESat-2), from the 2018-2019 cold season (Methods). The algorithm was applied to the inter-calibrated SSM/I and SSMIS Fundamental Climate Data Record^34^ over 1992-2020, from October to March. Later in the spring, the PMW signals are affected by snow melt^36^, and the accuracy of the PMW SIT is reduced.

**Figure 1:**
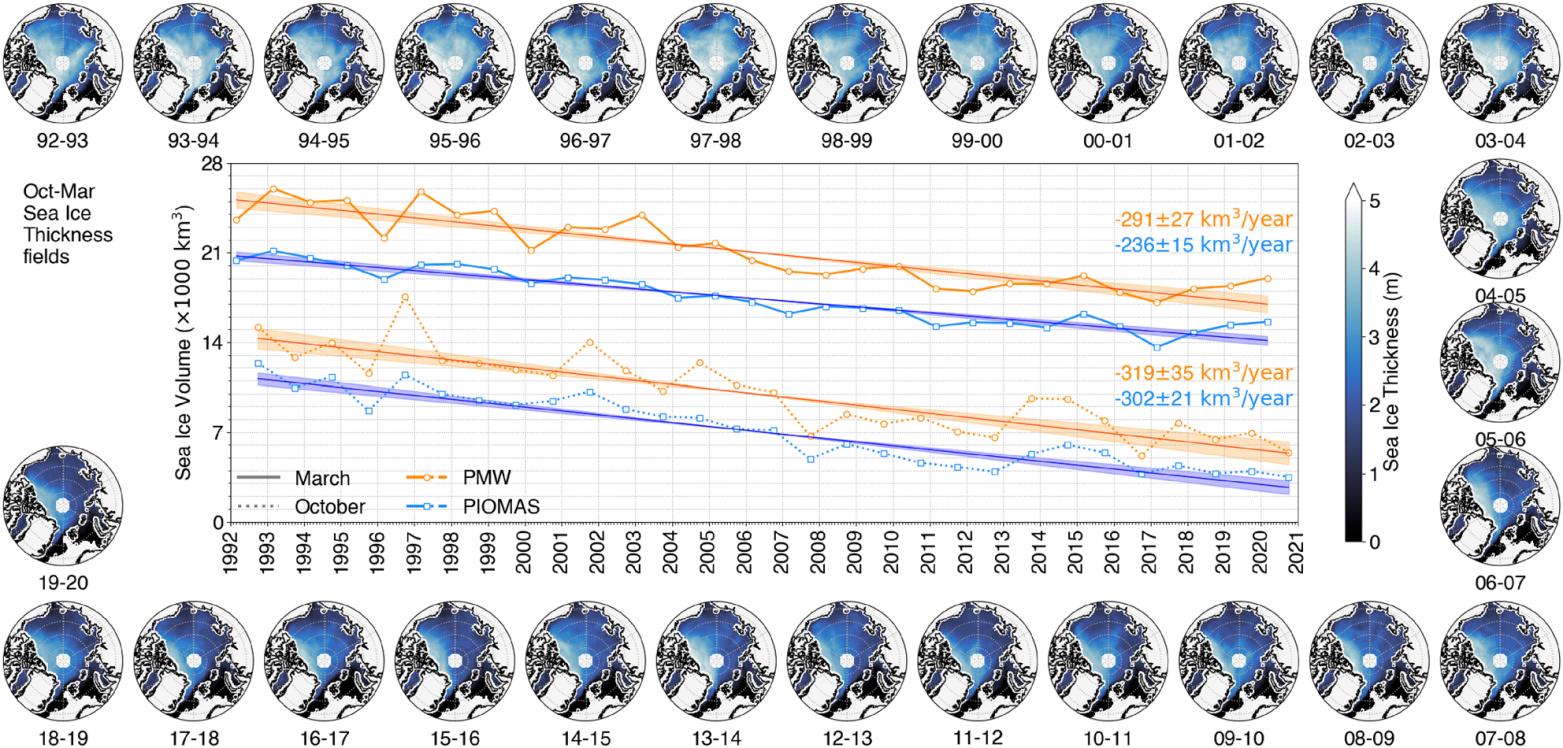
Decline of the Sea Ice Volume between 1992 and 2020 for October and March, estimated by Passive MicroWave (PMW) satellite observations and by the PIOMAS model, along with their associated linear trends. The maps show the average Sea Ice Thickness fields for every winter (Oct-Mar) in the period, as derived from the PMW observations.

PMW and PIOMAS SIT records have in common a poleward increase in thickness, relatively thin seasonal ice in marginal Arctic seas, and thicker multi-year ice mostly in the Arctic Basin (Fig. 1). Remarkably, SIT is on average 0.53 m higher in PMW than in PIOMAS, giving a corresponding SIV excess in PMW of 4615 ± 1852 km^3^ (Table 1). More detailed reasons for the SIT and SIV differences between the two datasets are given later in the text. PMW also features the pan-Arctic 1992-2020 sea ice thinning already documented in both observational datasets and model outputs including PIOMAS^13,14,37^, as maps shown in Fig. 1 indicate.

**Table 1:** Summary of 1992-2020 Sea Ice Volume statistics (Oct-Mar) for the PMW and PIOMAS time series. From left to right: mean SIV, mean SIV trend, standard deviation of monthly detrended anomalies, standard deviation of year-to-year change in monthly values, average Oct-Mar volume increase.

	$<SIV>\vert _{Oct-Mar}$	Trend $\vert _{Oct-Mar}$	$\sigma _{IA}$	$\sigma _{Y2Y}$	$\Delta SIV_{Mar-Oct}$
	[$10^{3} \hbox {km}^3$]	[$10^{3}\hbox {km}^3$/yr]	[$10^{3} \hbox {km}^{3}$]	[$10^{3}\hbox {km}^3$]	[$10^{3}\hbox {km}^{3}$]
PMW	17.5 ± 5.0	0.31 ± 0.03	1.42	1.81	10.8 ± 0.9
PIOMAS	12.6 ± 4.3	0.27 ± 0.02	0.76	0.92	10.1 ± 0.9

**Figure 2:**
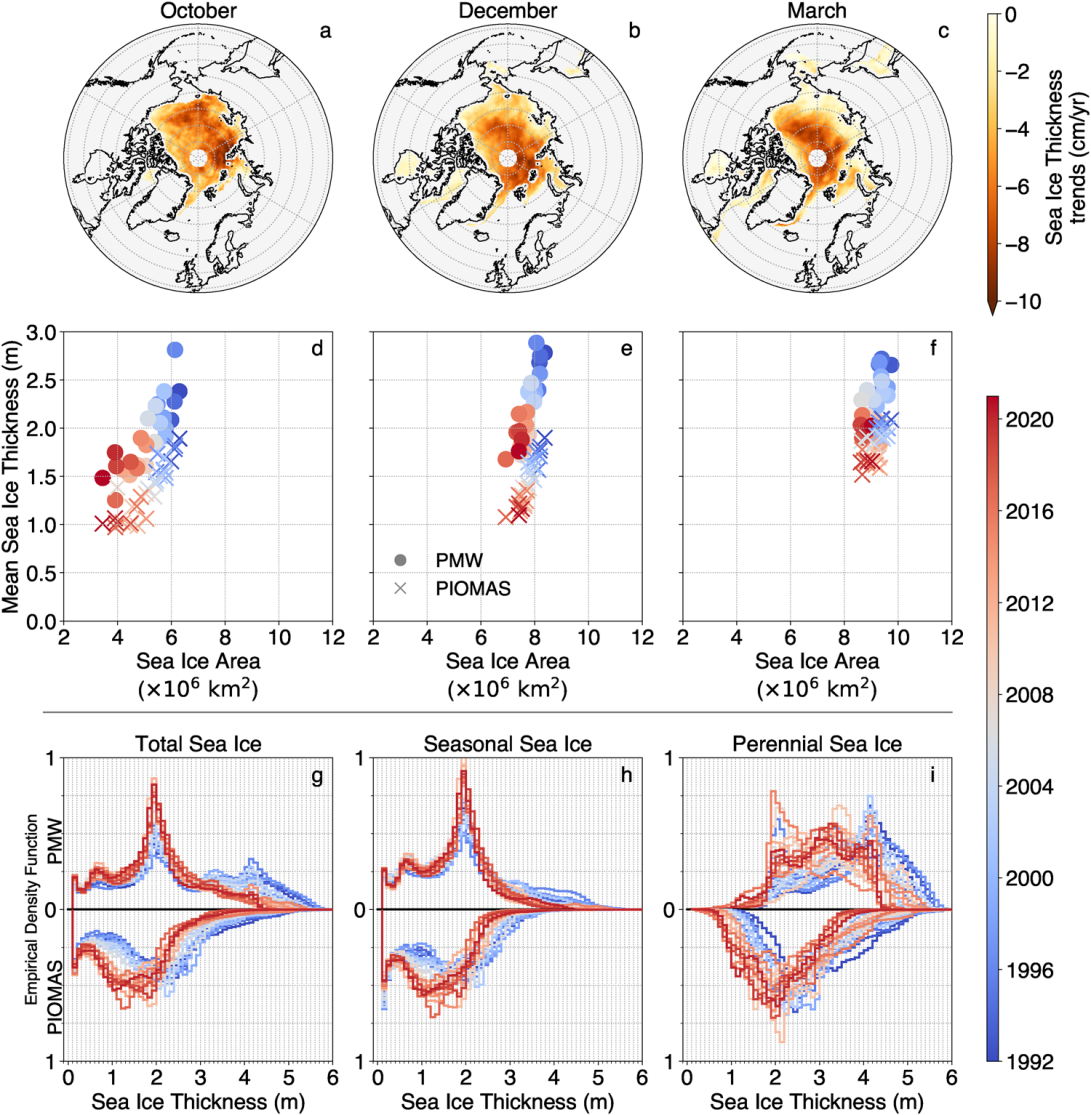
Changes in Sea Ice Thickness (SIT) between 1992 and 2020. Upper panels: Spatial distribution of the PMW SIT trends for October (**a**), December (**b**), and March (**c**) (with *P*-values  $\ll$ 0.05). Middle panels: Evolution of the average SIT and SIA for the whole Arctic during October (d), December (e), and March (f) between 1992 and 2020 for PMW (dots) and PIOMAS (cross). Lower panels: Evolution of the SIT distribution between 1992 (blue) and 2020 (red) over (**g**) the whole Arctic, (**h**) over seasonal sea ice, and (**i**) over perennial sea ice (distributions of the PMW SIT in the upper half of the figures and PIOMAS SIT in the lower half). SIT are binned over 10 cm.

The Arctic SIT reduction is largely consistent in PMW and PIOMAS datasets. In both, sea ice thinning is widespread, all-month, and strongest in the Central Arctic (Fig. 2, upper panels). Also, thinning trends are largest where the ice is the thickest. Indeed, the thick sea ice that was systematically found in the Central Arctic and regularly located on the Siberian Shelf in the 1990s, has become rare in recent years and confined to areas north of the Canadian Arctic Archipelago and north of Greenland. Quantitatively, over 2016-2020, the total area where SIT is above the third quartile (3.3 m in PMW and 2.4 m in PIOMAS) was drastically reduced as compared with 1992-1996 – by a factor of 3.5 in PMW (4.5 in PIOMAS). The rapid thinning of thick ice is consistent with a thermodynamic response to thermal forcing perturbations, stemming from the sea ice growth-thickness relationship^38^. Seasonally, thinning trends are largest in October, reaching up to 9 cm/year in the Eastern Arctic Basin and on the Siberian Shelf in PMW whereas PIOMAS thinning trends reach 8 cm/year in the Central Arctic and North of Greenland (Fig. 2, upper panels), consistently with the effects of late sea ice advance largest in early fall^39,40^.

PMW and PIOMAS are also highly consistent in terms of SIV changes ($r^2>0.9$ for most months, Table 2), and this despite their differences in mean SIV. A mostly linear decline is identified for all investigated months, with an Oct-Mar mean SIV trend of 312 km^3^/year in PMW, 17% larger than in PIOMAS (Table 2). In both records, SIV trends are larger early in the ice season (Oct-Dec) than in March, in agreement with previous findings^11^. We note, however, that the seasonal decrease in trend magnitude from October to March is more than 2 times larger in PIOMAS than in PMW. SIV trends are also largely sensitive to the period of interest: both PMW and PIOMAS agree on larger trends in the 2000-2010 decade and smaller trends over 2010-2020 (not shown) and trend can vary by 20-30% by changing the period of interest. Finally, inter-annual SIV variations are found in both records.

**Table 2:** Statistics of PMW and PIOMAS SIV time series. Trends ± uncertainties are given for each month between October and March.

	PMW trend (− 1) [km^3^/yr]	PIOMAS trend (− 1) [km^3^/yr]	PMW-PIOMAS trend difference [km^3^/yr]	PMW vs PIOMAS*r*^2^
Oct	319 ± 35	302 ± 21	17 (5 %)	0.92
Nov	314 ± 33	284 ± 19	30 (10 %)	0.92
Dec	321 ± 36	267 ± 19	54 (18 %)	0.93
Jan	318 ± 35	255 ± 19	63 (22 %)	0.91
Feb	309 ± 36	250 ± 16	59 (21 %)	0.86
Mar	291 ± 27	236 ± 15	55 (21 %)	0.94
Oct-Mar	312 ± 33 (11 %)	266 ± 17 (6%)	46 (17 %)	0.91

*r*^2^is obtained from a linear fit between monthly mean PMW and PIOMAS SIV time series.

### PMW and PIOMAS agree on the key impact of perennial ice loss on SIV trends, not on how much

In general, the patterns of thinning and volume loss are comparable in both PIOMAS and PMW datasets, even if the datasets result from different paradigms (the first is a combination of model calculations and data assimilation, and the second is based on a statistical PMW SIT retrieval trained on lidar altimetry). However, discrepancies exist between these records, warranting a more in-depth investigation to better characterize these differences. The primary cause of SIV change in both records is mean SIT, rather than Sea Ice Area (SIA), as illustrated in the middle panels of Fig. 2. From November to March, thinning contributes to 92% of the observed changes in SIV, while shrinking accounts for the remaining 8% (Fig. 2). In October, the SIA loss associated with late advance^39,40^ also contributes to 34% of SIV changes, whereas the remaining 66% can still be attributed to SIT. Hence, the SIV trends differ among PMW and PIOMAS most likely due to SIT differences, which we now further investigate.

Ice thickness changes are readily observable when organized into thickness categories (Fig. 2g). As expected, both PMW and PIOMAS feature significant amounts of ice thicker than 3 m during the 1990s (blueish curves, Fig. 2g) that is much less prevalent in the 2010s (reddish curves, Fig. 2g). However, we now see that the PMW-PIOMAS broad consistency does not hold as much in thickness space. In PMW, consistently with thicker ice, the thick ice peak is more developed than in PIOMAS. Also, the shape of the SIT distribution differs among the two sources: in PMW it is multimodal (with two to four peaks depending on the years) whereas in PIOMAS it has only two modal values. Thinning is also distributed differently across thickness categories. In PMW, thick ice drastically decreases in prevalence whereas thin ice becomes more likely, with no change in modal thickness (2.1 m), suggesting a replacement of thick ice by thin ice. In PIOMAS, next to the loss of the thickest ice, the whole distribution sweeps towards thinner ice, including a decrease in modal thickness.

Understanding the PMW-PIOMAS SIT differences benefits from splitting the SIT distribution over seasonal and perennial ice (Methods, and Fig. 2, panels h and i), which emphasizes issues for the latter ice type, in particular. Perennial, or multi-year ice, refers to sea ice that survives summer melt. In the Arctic, perennial ice tends to be thicker than younger seasonal ice, and has experienced significant losses over the past few decades. Perennial and seasonal ice can be identified on a yearly basis by analyzing the seasonal cycle of SIC, typically by counting the number of ice-free months^40^ or above a threshold as used here (Methods). We applied this diagnostic approach to PMW and PIOMAS records, utilizing PMW SIC records (Methods) and find that perennial ice is, on average, 89 cm thicker in PMW compared to PIOMAS. This discrepancy is greater than for the mean (53 cm) or for seasonal ice thickness (40 cm).

**Table 3:** Decomposition of Oct-Mar SIV budget (1992–2020) into four seasonal and perennial ice shrinking and thinning contributions.

Product	$\frac{dSIA_S}{dt}\cdot SIT_S$ Seasonal ice area change [km^3^/yr/yr]	$SIA_S\cdot \frac{dSIT_S}{dt}$ Seasonal icethinning [km^3^/yr/yr]	$\frac{dSIA_P}{dt}\cdot SIT_P$ Perennial ice area change [km^3^/yr/yr]	$SIA_P\cdot \frac{dSIT_P}{dt}$ Perennial ice thinning [km^3^/yr/yr]	$\Sigma$ terms [km^3^/yr/yr]	$\frac{dSIV}{dt}$ [km^3^/yr/yr]
PMW	42	− 59	− 225	− 67	− 309	− 312
PIOMAS	31	− 70	− 159	− 64	− 263	− 266
PMW-PIOMAS	11 (17%)	11 (33%)	− 65 (15%)	− 3 (4%)	− 46 (17%)	− 46 (17%)

Because of temporal averaging errors, the sum of terms is not rigorously identical to dSIV/dt.

What are the consequences of SIT differences on SIV changes? Said differently, how can SIV trends be relatively close despite SIT differences? To address this question, we decomposed SIV into seasonal (S) and perennial (P) contributions ($\mathrm {SIV= SIA_S\cdot SIT_S+SIA_P\cdot SIT_P}$) and analyze the contributions of area and thickness changes for each ice type (Table 3). In this framework, PMW and PIOMAS agree on the sign and relative importance of the SIV terms. Arctic SIV loss is dominated by perennial ice shrinking, with contributions from seasonal and perennial ice thinning.

The most important contribution to the difference in trend between PMW and PIOMAS SIV is, as expected, the loss of perennial ice volume due to shrinkage, which is 65 km^3^/yr greater in PMW than in PIOMAS. The perennial ice is thicker in PMW, so the loss of the same area of perennial ice (the same perennial ice shrinking rate $\mathrm {dSIA_P/dt}$ has been applied) has a greater impact in PMW than in PIOMAS. We note that 65 km^3^/yr is greater than the total trend difference between PMW and PIOMAS SIV. This is due to compensations, reducing the latter by about half. Firstly, seasonal ice thinning is less intense in PMW than in PIOMAS; secondly, the increase in SIV due to seasonal ice expansion is greater in PMW because seasonal ice is thicker in PMW than in PIOMAS. We also note that the SIC threshold used to delineate perennial and seasonal sea ice influences the budget decomposition, but not the PMW-PIOMAS differences.

The evolution of the SIT distribution over the years is also different in the two datasets. In PIOMAS, the changes in SIT are rather gradual, whereas PMW reveals a sharper transition (from the blueish to the reddish curves in Fig. 2i) around the year 2007, a well-documented minimum in Arctic sea ice extent^41^. In the Central Arctic region (Supplementary Information, Fig. S1), this rapid transition is particularly remarkable. Such abrupt change in SIT around 2007 has been reported in the Fram Strait from the 30-yr-long upward-looking-sonar record, which argues in favor of a regime shift^15^. One can speculate that PIOMAS, because it is a reanalysis product, is less capable of capturing abrupt mechanisms behind this regime shift.

### PIOMAS better captures mean thickness, PMW better retrieves variations

PMW and PIOMAS provide different SIT and SIV estimates. Can either be considered more reliable? To address this, PMW and PIOMAS SIT were colocated in time and space with available *in situ* SIT measurements, from Upward Looking Sonar (ULS) onboard submarines^19^.

*In situ* data do not distinctly favor either PIOMAS or PMW SIT as more realistic; PIOMAS better captures the mean SIT, while PMW better captures SIT variability. Indeed, over the three common periods of availability analyzed, PIOMAS tends to miss both the thinnest and thickest ice, whereas PMW shows occurrences of both, consistent with observations, but tends to produce thicker ice than observed (Supplementary Information, Fig. S2). More broadly, PMW appears to produce a more realistic multi-modal SIT distribution, consistent with various *in situ* sources, including airborne electromagnetic induction sounding surveys^42,43^, under-ice upward looking-sonar records^15^, and satellite-borne altimetry^44^. These findings are in line with previous evaluation exercises^12,14,31^. The assimilation in the PIOMAS reanalysis acts as a restoring term which could contribute to reduce the SIT variance in this dataset.

The origin of SIT biases in PMW and PIOMAS differs. In PMW, issues primarily stem from the availability of training data, with limited incorporation of data resembling the very thick ice conditions observed in the 1990s^31^. In this context, PMW can be considered reliable for ice <2 m, where the training dataset is providing enough data, but more uncertain for thicker ice, particularly for the oldest years in the record, where higher SIT are observed.

In PIOMAS, SIT biases arise from issues in model physics, forcing or assimilated data. As for PMW, the thinnest ice is the most likely to be well reproduced by the model, as sea ice thermodynamic processes have been studied for a long time^38,45,46^, and are rather well reproduced in large-scale sea ice models^47^. By comparison, the simulated ice drift and deformation are more uncertain^48^ and can have a large influence on sea ice via residence type and ridging^15,49^, posing more complex challenges than thermodynamics. Hence, significant SIT uncertainties may stem from model ice dynamics, particularly rheology^50^ and ridging schemes^51^, which remain subjects of long-standing debate and active research in the sea ice modelling community^52–54^.

In this line of thought, it is complicated to designate PIOMAS reanalysis or the PMW estimates as more realistic: only a few *in situ* data are available, and both sources have different and time-dependent issues. The origin of these problems is different, and in both cases, thick sea ice is more affected than thin ice.

### Sea ice volume inter-annual variability is in phase between PMW and PIOMAS

**Figure 3:**
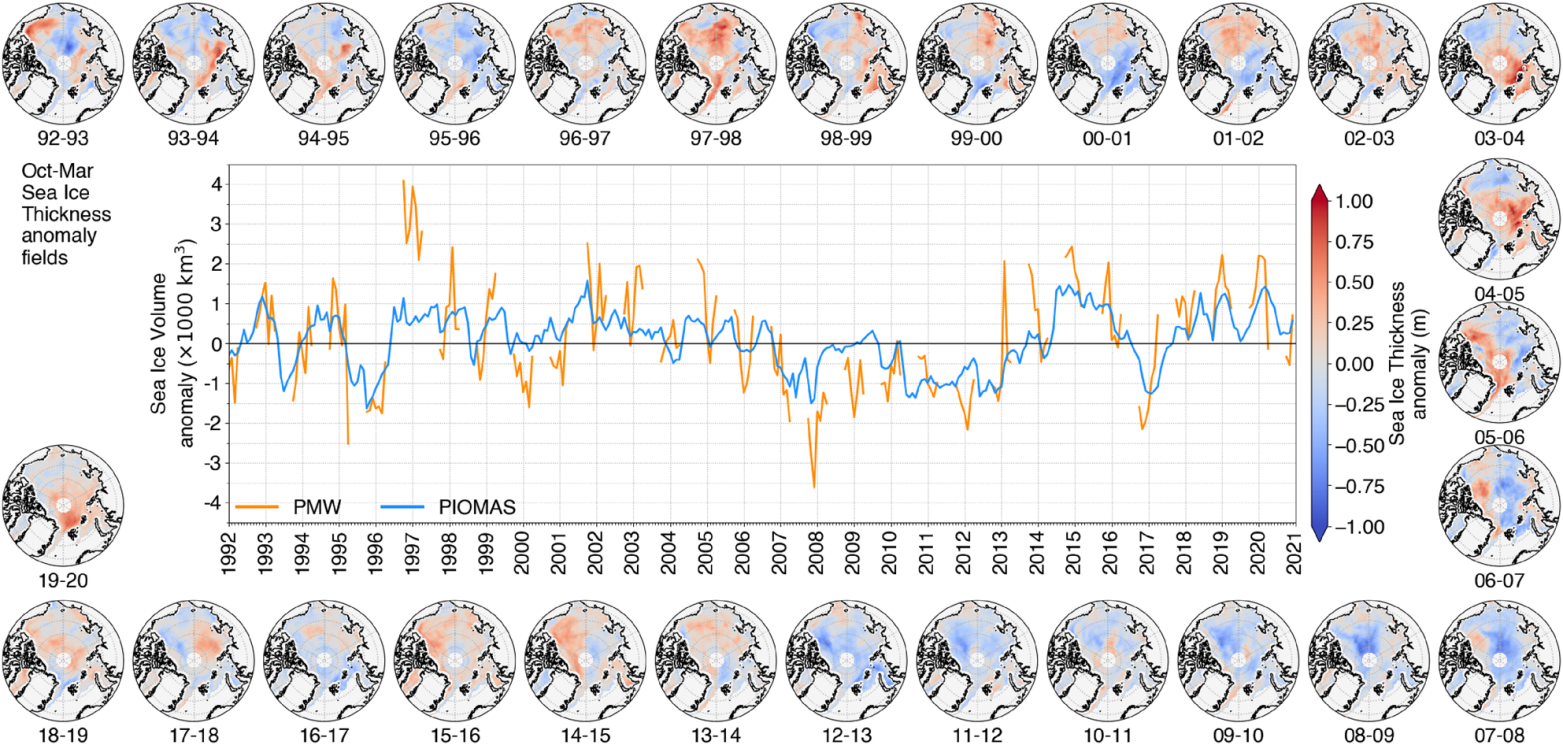
Detrended Sea Ice Volume Anomalies from PMW and PIOMAS. The maps show the detrended Sea Ice Thickness anomaly fields from PMW for every winter (Oct-Mar) in the period.

A final objective of our analysis is to examine how consistent PMW and PIOMAS are in terms of inter-annual SIV variability. Inter-annual SIV variations superimpose upon long-term trends in both products and Fig. 3 indicates many common features in the PMW and PIOMAS detrended anomalies (Supplementary Information, Fig. S3 also shows time series of year-to-year anomalies for each of the investigated months). Notably, PMW and PIOMAS generally agree on which years on record have high or low SIV, as evidenced by an $r^2$ value of 0.55 for detrended anomalies. The decomposition of SIV detrended anomalies into the contributions of the SIT and the SIC (Methods and Supplementary Information, Fig. S4) show that the SIT anomalies provide the dominant contribution to PMW and PIOMAS SIV anomalies, around six times higher than the absolute contribution from SIC anomalies for PMW (and five times for PIOMAS), similar from previous observations^55^. Inter-annual variations are also remarkably consistent in space (Supplementary Information, Fig. S5).

Inter-annual SIV variations are nonetheless larger in PMW than in PIOMAS: year-to-year relative differences in SIV are 1.9 times higher in PMW than in PIOMAS, and the standard deviation of detrended SIV anomalies is 2.7 times higher for PMW (Table 1). Yet both sources concur that variability in SIV is relatively higher than for SIA: the standard deviation of detrended October to March SIV anomalies is 8% of mean SIV for PMW and 6% for PIOMAS, which is 3-4 times larger than comparable values for SIA (< 2%). Both also agree on highest inter-annual variability occurring early in the ice season: year-to-year variations are almost three times larger in October than in March, for both PIOMAS and PMW (Supplementary Information, Fig. S3).

The anomaly time series emphasizes several events which occurred during the last decades that are also seen on Fig. 1. The early winter anomaly in polar winter 2006-2007 has already been documented from submarine measurements^15^, and it is associated to a low sea ice extent^41^. PMW and PIOMAS also feature the strong SIV increase in 2014, after a low melting in 2013, as measured with CryoSat-2^56^. The replenishment of multiyear sea ice in 2013 and 2014, north of Greenland and the Canadian Archipelago^57^ on the spatial fields of detrended SIT anomalies is confirmed for both PMW and PIOMAS (maps in Fig. 3 and in Supplementary Information, Fig. S6).

The analysis of individual years perhaps shows a limit of the PMW record. Indeed, the highest absolute PMW SIV anomaly of 4000 km^3^ which occurs during winter 1996–1997 is 3000 km^3^ higher than PIOMAS anomaly. In any case, this year seems outside the possible range of SIV, with a replenishment of nearly 6000 km^3^ between October 1996 and October 1997. PIOMAS also sees a positive anomaly but about half as large. Therefore, part of the anomaly might be realistic and related to the strong ENSO (El Niño Southern Oscillation) event that occurred around 1997^58,59^, as sea ice was reported to have been affected by the ENSO event^60,61^.

## Discussion and Conclusion

We present the first consistent multi-decadal (1992-2020) satellite-based time series of winter pan-Arctic SIT and SIV, covering up to 87.6 °N. These time series are derived from inter-calibrated SSM/I- SSMIS, using an algorithm trained on a cold season of Arctic lidar altimetry-derived SIT. We compare the new PMW SIT retrieval dataset with PIOMAS, a reference SIT product, based on an ice-ocean model forced by atmospheric reanalyses and assimilating PMW sea ice concentration^35^.

Despite relatively large mean state SIV differences (4.6 × 10^3^ km^3^,  $37\%$), we find many commonalities in the way the PMW SIT dataset and PIOMAS represent Arctic SIV and its changes. The PMW and PIOMAS linear SIV trends are $0.27 - 0.31 \times 10^3$ km^3^/yr, only differing by 46 km^3^/yr (17%) on average. Inter-annual SIV variations are consistently phased, and represent 7-11% of the mean. Finally, both products indicate the preferential loss of thick ice, and largest SIT trends in October.

These differences in volume and mean trends between the two datasets are substantially lower than current uncertainty estimates. 4.6 × 10^3^ km^3^ is larger than the 1.35 × 10^3^ km^3^ estimated for PIOMAS^12^, but much smaller than the inter-model SIV spread from model-based reanalyses (> 10 × 10^3^ km^3^,^13^) retained in the IPCC last assessment^62^. Furthermore, the PMW-PIOMAS SIV trend differences (30 km^3^/yr) are less than 3 times smaller than uncertainties evaluated for PIOMAS (100 km^3^/yr,^12^) thus an order of magnitude lower than inter-model spread found among reanalysis systems (526 km^3^/yr,^13^). That high and low SIV years are consistent between PIOMAS and PMW can also be seen as positive, as it is not the case for reanalysis systems^13^.

Such comparatively low PMW-PIOMAS differences in SIV mean and trends stem from consistent representation of key SIV contributors in the two products, namely seasonal and perennial ice coverages, seasonal ice thickness, and the fact that both products also capture the thickness contrast between relatively thin seasonal and relatively thick perennial ice. However, perennial ice thickness differences (89 cm on average) remain the largest uncertainty source, both for mean SIV and SIV trends. This higher uncertainty in SIT can be due to the lack of high SIT in the training database for the PMW retrieval, as well as to the more complex physics of perennial ice in models for PIOMAS. In contrast, we argue that as seasonal sea ice is thin, its thickness can be retrieved from data with increased confidence^31^, and PIOMAS faithfully analyzed the well-known seasonal thermodynamic growth.

It is also noteworthy that key features of the long-term SIV changes found in both PMW and PIOMAS are physically consistent. That thick ice is preferentially lost stems from basic sea ice thermodynamics^38^. The reduced SIV trend over the last decade has been explained as a consequence of negative feedbacks provided by increased growth and reduced SIV export due to sea ice thinning^63^. Largest trends in beginning of winter can be seen as a consequence of later advance^39,40^.

Based on the above arguments, we first defend that Arctic SIV uncertainties are not as high as the spread in Arctic sea ice model reanalyses suggests. This considered, SIV uncertainties are probably overestimated in sea ice model reanalysis, in particular when including simulations with no use of data assimilation. By contrast, PIOMAS and PMW could be considered as more reliable references, although neither PMW nor PIOMAS should be considered as absolute truth. PMW suffers from an identified thick bias for oldest perennial ice on record, whereas PIOMAS is known to underestimate SIT variance. Second, we argue that the analysis presented in this paper shows that long-term PMW time series includes useful SIT information. Therefore, the incorporation of these PMW SIT and SIV retrievals into model simulations could benefit to Arctic sea ice reanalyses (such as PIOMAS) or seasonal predictions^64^, as the spatial and temporal coverage of the PMW product is well suited for the assimilation^65^.

Improving our understanding of long-term changes in Arctic sea ice thickness and volume could benefit from PMW contribution. Overall and despite its limitation, our PMW record can provide important progress on the understanding of SIT and SIV pan-Arctic changes, particularly over the long time scales analyzed here that are not available from other satellite-based observations. Next to the essential improvement of altimetry-based SIT products, more research should be conducted to explore the potential of PMW for SIT and SIV. As the PMW SIT skill is not fully expected from physical principles^66^, the indirect links between the PMW and the SIT should be investigated (e.g., ice salinity, microstructure in the volume, surface roughness). It could be worth acquiring PMW observations at intermediate scales (between satellite and *in situ*) in order to understand the effects of pressure ridges or rubble fields on PMW signals, which are currently difficult to envision. The Copernicus Microwave Imaging Radiometer (CIMR^67^) and the Copernicus Polar Ice and Snow Topography Altimeter (CRISTAL^68^), both planned to be launched at the end of the 2020s, are primarily designed to observe the polar regions in support of the Integrated European Policy for the Arctic. CIMR will provide passive microwave observations, with unprecedented accuracy and spatial resolution (5 km at 18 and 36 GHz used in this study). Exploiting the synergies between CIMR and CRISTAL passive and active microwave observations is strongly encouraged, for an improved quantification of the sea ice thickness and volume changes in the future.

## Methods

### Data

#### The SSM/I and SSMIS passive microwave observations

The Special Sensor Microwave / Imagers (SSM/I) and the Special Sensor Microwave Imager Sounder (SSMIS) sensors, on board the USA Defense Meteorological Satellite Program (DMSP) span more than 30 years of passive microwave observations, with almost global coverage of the Arctic (up to 87.6° N). These instruments acquire brightness temperatures at different microwave frequencies, including the 18 and 36 GHz that are used here to calculate the SIT, and then the SIV. The change from the SSM/I series to the SSMIS corresponds to a significant modification of the instrument, with the addition of sounding channels as well as different observation strategies. The observations from the multiple instruments over time have been carefully intercalibrated so they can be used for climate applications^34^. The corresponding datasets used in this study (from the satellites F11, F13, F14, F15, F16, F17 and F18) are available from the Climate Satellite Application Facility at EUMETSAT. The brightness temperatures have been gridded on an equal area grid at 12.5 km resolution, the same as the grid used by the OSI-SAF for the SIC^69^.

#### PIOMAS

The Pan-Arctic Ice-Ocean Modelling and Assimilation System (PIOMAS) combines a coupled sea-ice-ocean with observations using data assimilation. Satellite-derived Sea Ice Concentration (from passive microwaves) as well as Sea Surface Temperature (from infrared) are assimilated into PIOMAS, to provide an alternative approach to estimate regional trends in volume^35^. It has been designed to estimate Sea ice Thickness and then Sea Ice Volume (among other climate variables) since the beginning of sea ice satellite observations. Here, we use the 2.1 version of the PIOMAS^12^ from 1992 to 2020. PIOMAS is formulated in a generalized orthogonal curvilinear coordinate (GOCC) system and is here projected on the Northern EASE-Grid 2.0 at 12.5 km resolution for better comparison with the other datasets used in this study.

### In situ measurements

The sea ice thickness climate data record (sea ice CDR) of *in situ* observations of ice draft and thickness^70^ integrates measurements from submarine Upward Looking Sonar (ULS) from U.S. submarines^71^. Only segments acquired between October and March, up to 87.6° N are kept.

A total of 3 months are providing temporally and spatially collocated *in situ* measurements, i.e. October 1996 and 2000, and November 2005. All of them have been acquired in the Central Arctic. Data from the ULS have been reprojected on the Northern EASE-Grid 2.0 at 12.5 km resolution and averaged on the same temporal window for better comparison with the PMW and PIOMAS data.

### Sea Ice thickness product from passive microwave radiometers

Sea Ice Thickness is retrieved from brightness temperatures acquired by the SSM/I and SSMIS passive microwave radiometers. The methodology has been described in detail^31^, here we summarize the theoretical basis of the algorithm. The algorithm uses the statistical relationships observed between passive microwave observations and SIT to train a Neural Network (NN) to reproduce ICESat-2 SIT from brightness temperatures at 18 and 36 GHz. The NN has been trained on the polar winter 2018-2019 where it showed good performance when compared to the CryoSat-2 satellite retrieval and the Operation Ice Bridge airborne measurements^31^. NNs have already been widely used in satellite remote sensing for the retrieval of numerous geophysical parameters, including sea ice variables^32,72,73^. We adopted a classic NN architecture called Multi Layered Perceptron (MLP)^74^ to establish the non-linear relationship between the observed brightness temperatures and SIT. This MLP architecture is appropriate to approximate multivariate non-linear relationships^75–77^, and is being applied to build the statistical model reproducing the mapping between brightness temperatures and SIT. The ICESat-2 SIT is expected to be independent of passive microwave observations^23^ and is retrieved from a different frequency domain (visible versus microwave)^78^.

### Sea Ice volume

The SIV represents the spatial integration of the SIT field over the area $\textrm{dA}$ of each grid cell. Here we integrated the SIT over the Sea Ice Area (SIA), defined as the area where the Sea Ice Concentration (SIC) is $\ge ~\mathrm {15~\%}$, taking into account the SIC for each location:

1 $$\begin{aligned} \textrm{SIV} = \mathrm {\sum _{SIA} SIT \cdot SIC~dA} \end{aligned}$$

The SIC is provided from EUMETSAT Ocean and Sea Ice Satellite Application Facility (OSI SAF)^6^. The EASE Grid 2.0 at 12.5 km of resolution being an equal area grid, each cell represents the same area, here 12.5 km × 12.5 km.

### Seasonal and perennial sea ice

The SIC is also used to discriminate between seasonal and perennial sea ice: pixels with SIC greater than 80% year-round are considered as perennial ice. This simple yet efficient method does not rely on some other products than SIC also estimated from PMW brightness temperatures, but does not take into account the drift of sea ice over the year.

### SIV anomalies

SIV anomalies, SIV’, are obtained from the time series of pan-Arctic SIV by removing the 1992-2020 trend for each month. The SIV anomalies can also be decomposed as follows^55^:

2 $$\begin{aligned} \mathrm {SIV'} = \mathrm {\sum _{SIA} (SIC'\cdot {\overline{SIT}} + {\overline{SIC}}\cdot SIT' + SIC'\cdot SIT')~dA} \end{aligned}$$

where primes represent the anomalies of SIC and SIT, bars represent the untrended climatology, and A represent the area.

## Supplementary Information

Supplementary Figures.

## Data Availability

The datasets generated during and/or analysed during the current study are available from the corresponding author on reasonable request.
