# Author Correction: Winter arctic sea ice volume decline: uncertainties reduced using passive microwave-based sea ice thickness

**DOI:** 10.1038/s41598-024-77046-w

**Published:** 2024-10-28

**Authors:** Clement Soriot, Martin Vancoppenolle, Catherine Prigent, Carlos Jimenez, Frédéric Frappart

**Affiliations:** 1 LERMA, Observatoire de Paris, CNRS, Université PSL, Paris, France; 2 Centre for Eath Observation Science, University of Manitoba, Winnipeg, MB Canada; 3 Sorbonne Université, Laboratoire d’Océanographie et du Climat, CNRS/IRD/MNHN, Paris, France; 4 Estellus, Paris, France; 5 ISPA, UM 1391, INREA/Bordeaux SCience Agro, Villenave, d’Ornon 33140 France

Correction to: *Scientific Reports* 10.1038/s41598-024-70136-9, published online 09 September 2024

In the original version of this Article Carlos Jimenez was incorrectly affiliated with ‘Centre for Earth Observation Science, University of Manitoba, Winnipeg, MB, Canada.’ Their correct affiliations are listed below.

1 LERMA, Observatoire de Paris, CNRS, Université PSL, Paris, France.

4 Estellus, Paris, France.

Furthermore, the Abstract contained an error.

“We find many commonalities in Arctic SIV changes from a PMW sea ice thickness (SIT) 1992-2020 time series reconstructed with a neural network algorithm trained on lidar altimetry, and the reference PIOMAS reanalysis: relatively low differences in SIV mean (4615 km^3^, 37%), SIV trends (46 km^3^, 17%), and phased variability (r^2^=0.55).”

now reads:

“We find many commonalities in Arctic SIV changes from a PMW sea ice thickness (SIT) 1992-2020 time series reconstructed with a neural network algorithm trained on lidar altimetry, and the reference PIOMAS reanalysis: relatively low differences in SIV mean (4615 km^3^, 37%), SIV trends (46 km^3^/yr, 17%), and phased variability (r^2^=0.55).”

The original Article has been corrected.

