# Supplementary material for: Winter arctic sea ice volume decline: uncertainties reduced using passive microwave-based sea ice thickness
Source: Sci Rep. 2024 Sep 9;14:21000. doi: 10.1038/s41598-024-70136-9 (PMC11383946; doi:10.1038/s41598-024-70136-9)
Supplement: Supplementary file 1 — Supplementary Figures. [file 41598_2024_70136_MOESM1_ESM.pdf]

Supplementary Information

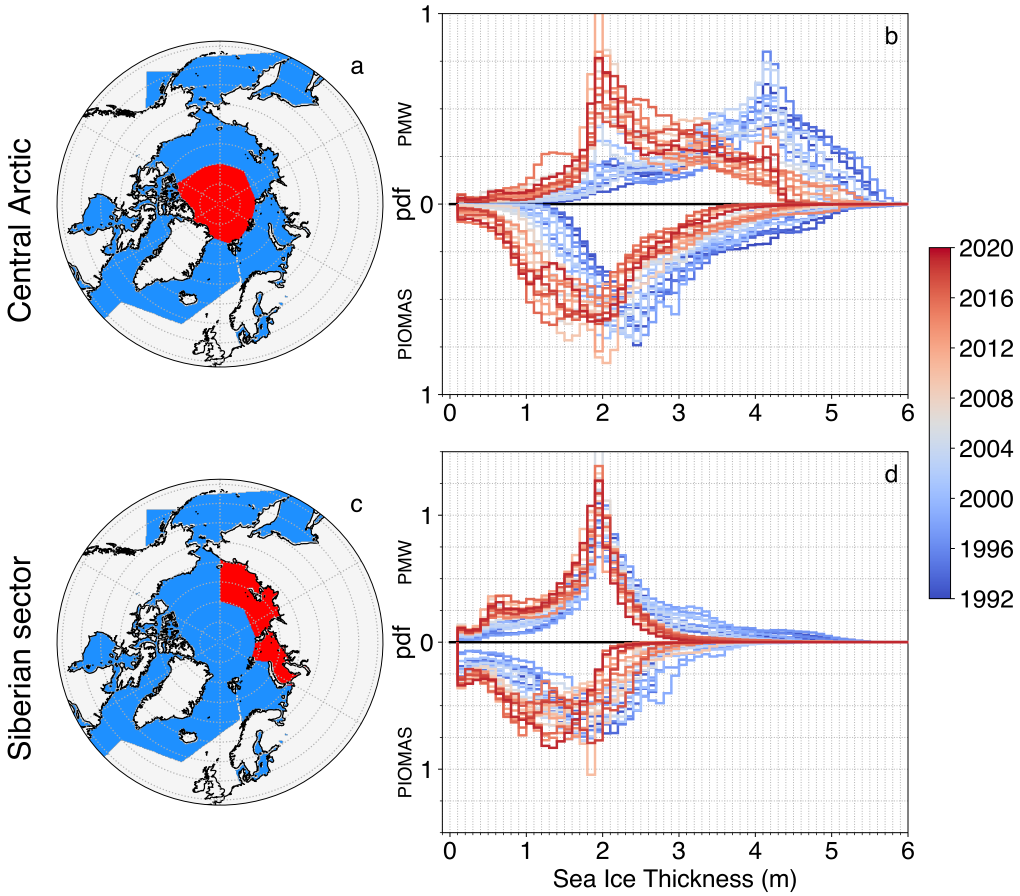

Figure S1. Examples of regional temporal evolutions of Sea Ice Thickness (SIT) distributions between 1992 and 2020 for PMW (upper distributions) and PIOMAS (lower distributions). Upper panels: Central Arctic mask (a) and the associated SIT distribution (b). Lower panels: Siberian sector mask, including the East Siberian Sea, the Kara Sea and the Laptev Sea (c) and the associated SIT distribution (d).

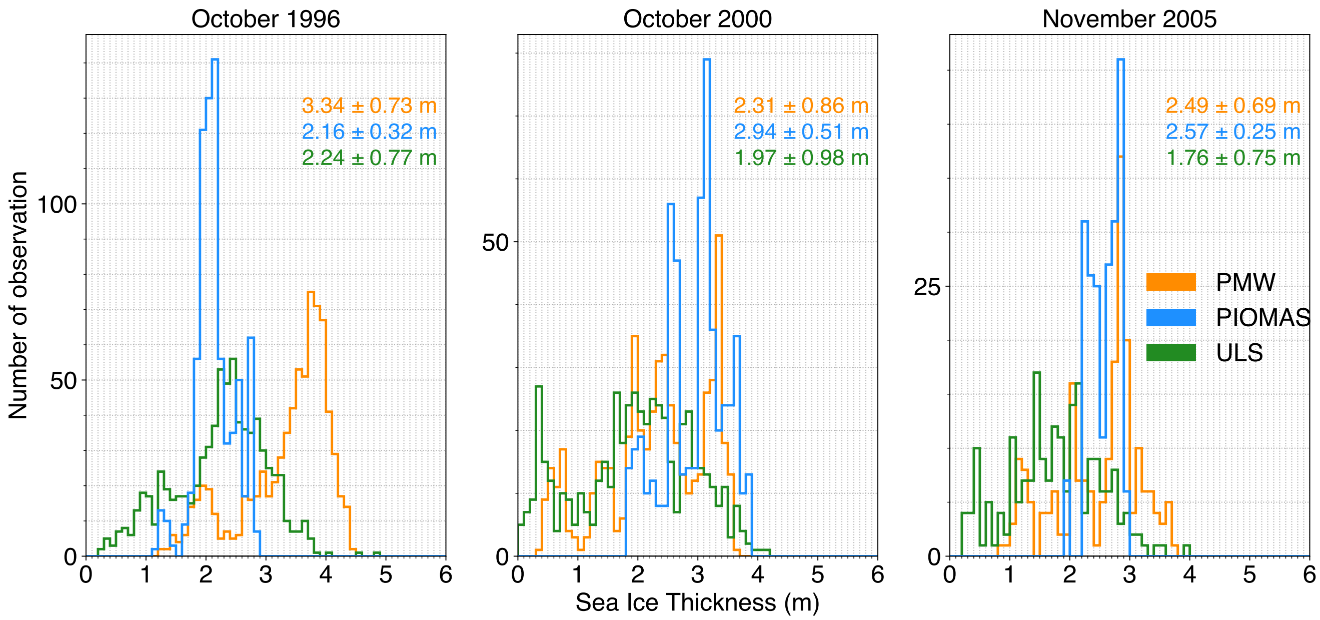

Figure S2. Sea Ice Thickness (SIT) distribution for PMW (orange), PIOMAS (blue) and submarine ULS (green) 3 months. For each month, the mean and the standard deviation of each distribution is given. SIT are binned over 10 cm.

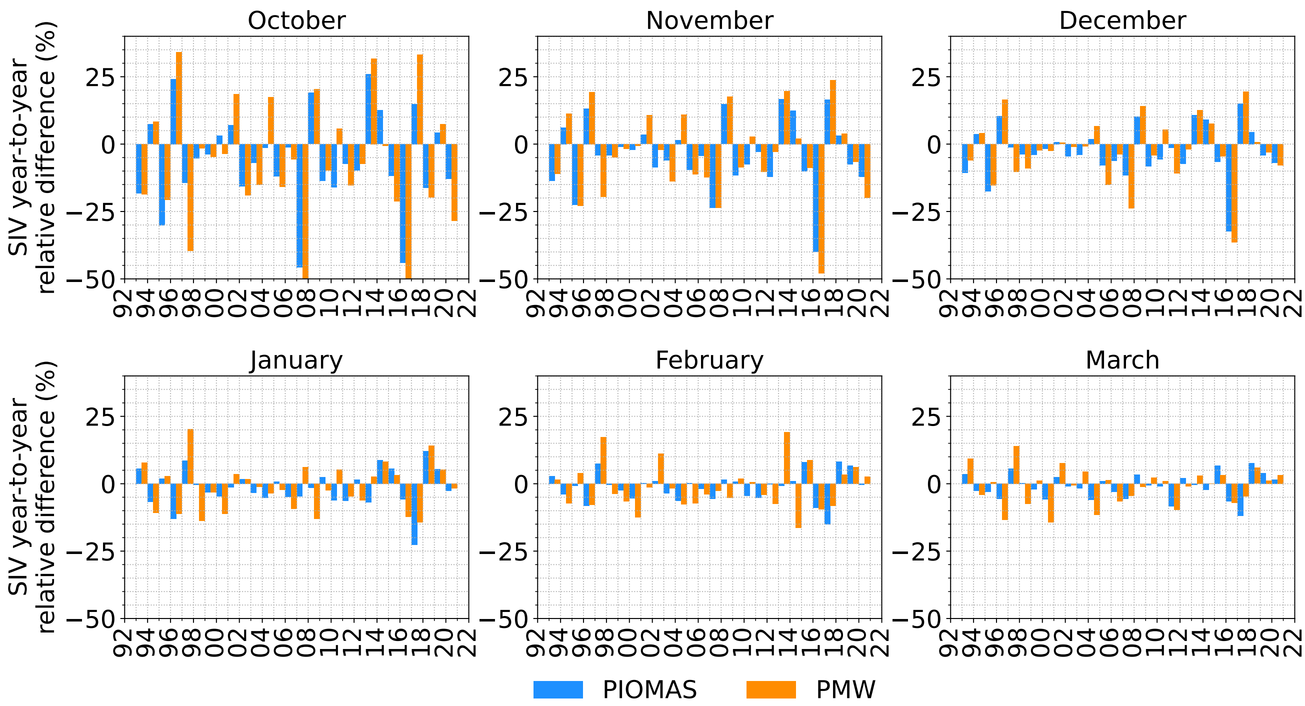

Figure S3. Sea Ice Volume year-to-year change (relative difference, in percent from the SIV of the previous year) for each month between October and March.

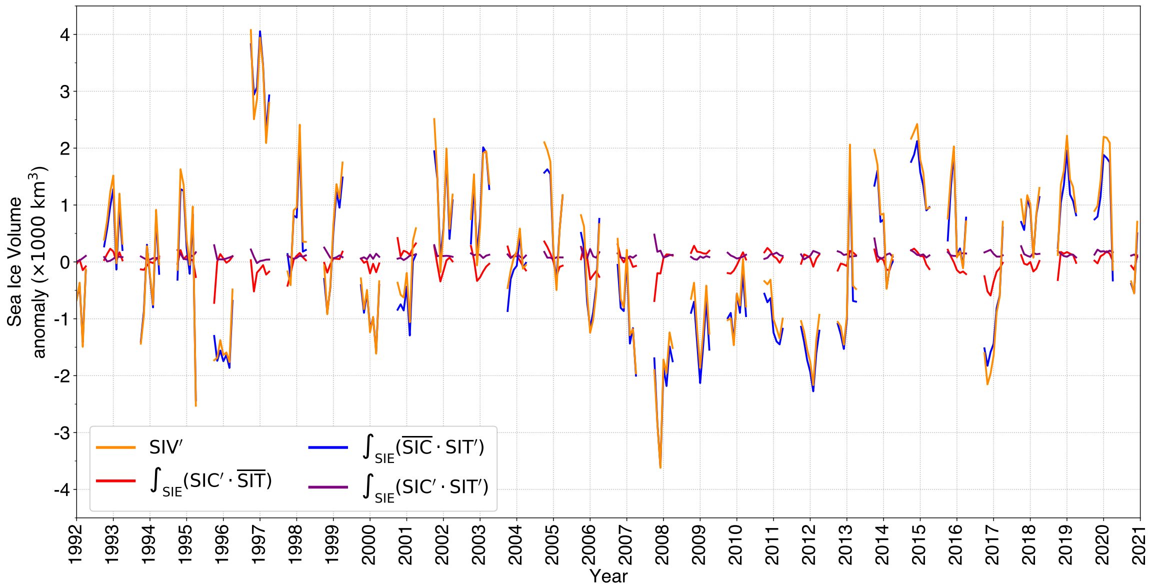

Figure S4. PMW Sea Ice Volume (SIV) anomaly decomposition as described in equation **??**. In orange, the SIV anomaly after removing the climatological seasonal cycle of SIV obtained from the 1992–2020 time series of SIV from PMW SIT and OSI SAF SIC. It is the same SIV anomaly as presented in orange in Figure **??**. In red, blue, and purple are the contributions of SIC anomalies, SIT anomalies, and their correlated components, respectively, to the time series of SIV anomalies. The correlations between the anomalies of SIV with respective anomalies of SIT, SIC, and their correlated components, are 0.99, 0.30, and -0.04. SIT anomalies provide the dominant contribution to SIV interannual variability compared to SIC anomalies.

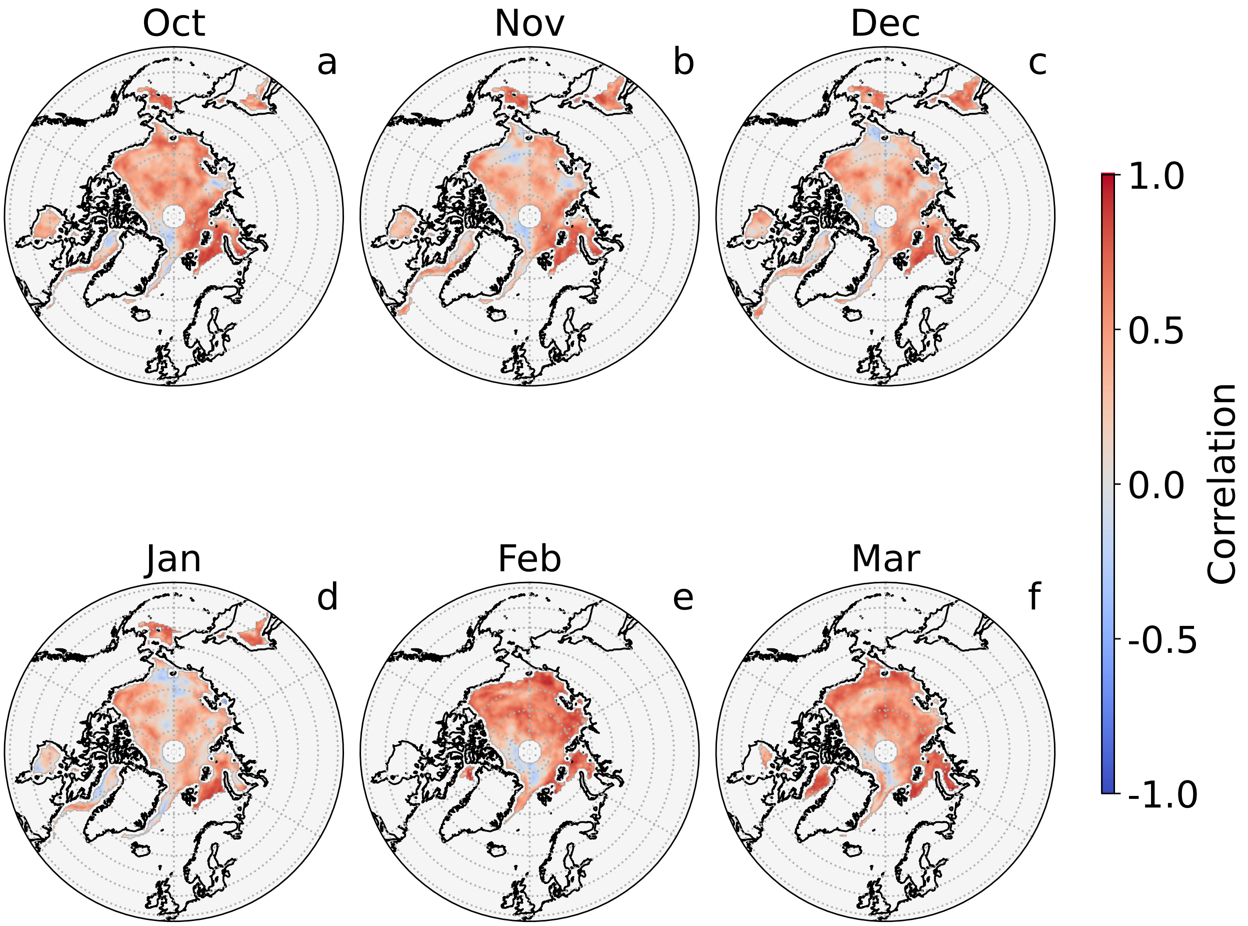

Figure S5. Maps of temporal linear correlation (r) of yearly detrended Sea Ice Thickness anomalies between PMW and PIOMAS for each month between October and March.

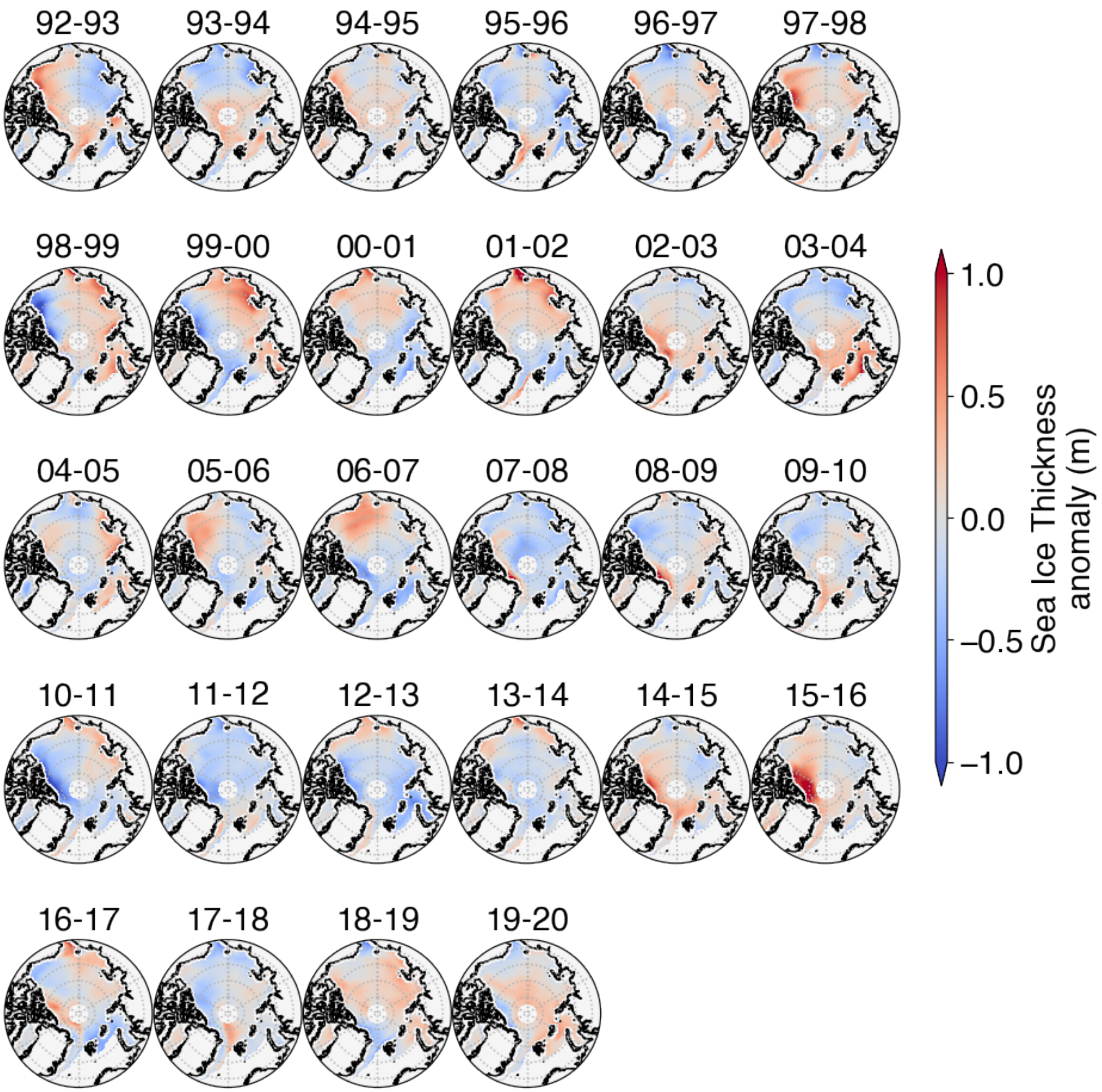

Figure S6. Maps of detrended and seasonally adjusted Sea Ice Thickness anomaly fields from PIOMAS for every winter (Oct-Mar) in the period.
